# Routine Tumor Testing for Homologous Recombination Deficiency in Patients With High Grade Epithelial Ovarian Cancer at a Statewide Gynecological Cancer Service in Western Australia: An Observational Study

**DOI:** 10.1002/cnr2.70335

**Published:** 2025-09-02

**Authors:** Kaamini Planisamy, Elena Ctori, Benhur Amanuel, Emma R. Allanson, Chloe Ayres, Martin Buck, Giselle Howard, Yee Leung, Tarek Meniawy, G. Raj K. A. Mohan, Cassandra B. Nichols, Navin David Pallayoor, Paul A. Cohen

**Affiliations:** ^1^ Western Australia Gynaecological Cancer Service, King Edward Memorial Hospital Subiaco Western Australia Australia; ^2^ PathWest Molecular Anatomical Pathology, Sir Charles Gairdner Hospital, Hospital Avenue Nedlands Western Australia Australia; ^3^ Division of Obstetrics and Gynaecology Medical School, University of Western Australia Crawley Western Australia Australia; ^4^ Queen Elizabeth II Cancer Centre, Sir Charles Gairdner Hospital, Hospital Avenue Nedlands Western Australia Australia; ^5^ Genetic Health Western Australia, King Edward Memorial Hospital Subiaco Western Australia Australia

**Keywords:** homologous recombination deficiency (HRD), HRD testing, ovarian cancer, poly‐ADP ribose polymerase inhibitors

## Abstract

**Background:**

Poly‐ADP ribose polymerase inhibitors have been shown to improve progression‐free survival in patients with advanced high‐grade epithelial non‐mucinous ovarian cancers characterized by a deficiency in homologous recombination (HRD). Guidelines recommend all patients with advanced high‐grade epithelial ovarian cancer undergo genomic tumor testing for HRD. Our aim was to evaluate the first year of HRD testing at the statewide Western Australia Gynecologic Cancer Service to assess factors associated with obtaining a diagnostic HRD testing result.

**Methods:**

Retrospective chart review.

**Results:**

HRD testing was indicated in 84 patients, and ordered in 79, of which three had non‐diagnostic/inconclusive results, all due to insufficient tumor quantity. One patient had the sample collected using a 20‐gauge core biopsy needle under image guidance, one patient following interval debulking surgery, and one following primary debulking surgery. Of 76 patients with an HRD result, HRD was positive in 29 (38.2%). A somatic *BRCA* mutation was detected in six of these 29 patients (20.6%) and HRD positive, BRCAwt was detected in 23 of 29 patients (79.4%). All core biopsies with 16‐ and 18‐gauge needles had a diagnostic HRD result. Ten of 11 patients who were treated by neoadjuvant chemotherapy and whose biopsies were obtained at interval cytoreductive surgery had sufficient tumor tissue for testing and had a diagnostic HRD result. All ascitic/pleural fluid samples sent for HRD testing yielded diagnostic results.

**Conclusions:**

Compliance with HRD testing was high, and only three of 79 (3.8%) patients had non‐diagnostic results.

## 1 Introduction

Ovarian cancer is the eighth most common cancer among women worldwide [1]. Approximately 90% of ovarian cancers are epithelial ovarian cancers, which are mostly asymptomatic in the early stage [2]. There is currently no effective screening test, and most patients present with advanced disease [3]. Despite initially responding to surgery and chemotherapy, approximately 80% of patients will relapse and die of their disease [4].

Approximately 50% of high‐grade epithelial non‐mucinous ovarian cancers are characterized by a deficiency in homologous recombination, a high‐fidelity DNA double‐strand break repair pathway that maintains genomic stability [5, 6]. The SOLO‐1 trial showed that first‐line maintenance therapy with Olaparib, an oral poly‐adenosine diphosphate‐ribose polymerase inhibitor (PARPi), significantly improved survival in patients with newly diagnosed advanced ovarian carcinoma that had germline or somatic *BRCA1/2* gene mutations [7]. The PRIMA trial showed that the PARPi Niraparib was associated with a significantly longer progression‐free survival compared to placebo in patients whose tumors were homologous recombination deficient (HRD) [8]. PARPi are small molecule inhibitors of PARP1/2 that are synthetically lethal to cells that are HRD. The PAOLA‐1 trial demonstrated a progression‐free survival benefit of first‐line maintenance Olaparib in combination with bevacizumab in patients with advanced ovarian cancer whose tumors were HRD and *BRCA* wild type (BRCA*wt*) [9]. This finding has allowed up to 50% of patients with advanced epithelial ovarian cancers to benefit from maintenance PARPi therapy. International guidelines now recommend all patients with advanced high‐grade epithelial ovarian cancer undergo genomic tumor testing for *BRCA* and HRD [10, 11]. In Australia, national guidance on HRD testing was published in September 2023 [12] when HRD testing was recommended for all patients with newly diagnosed high‐grade non‐mucinous epithelial ovarian, fallopian tube, and primary peritoneal carcinomas.

The aim of the current study was to evaluate the first year of HRD testing at the statewide Western Australia Gynecologic Cancer Service (WAGCS) to assess factors associated with obtaining a diagnostic HRD testing result. Secondary objectives were to determine the proportion of patients with high‐grade non‐mucinous epithelial ovarian cancers who received tumor HRD and germline genetic testing and to identify factors associated with non‐receipt of testing.

## 2 Materials and Methods

The term ‘ovarian cancer’ used throughout the manuscript refers to a malignancy originating in the ovaries, fallopian tubes, or peritoneum. The study was approved as an audit by King Edward Memorial Hospital, Subiaco, Western Australia (GEKO Quality Improvement system reference 54 758, 27 September 2024). Patients provided informed consent for HRD testing and germline genetic testing as part of routine clinical care.

Inclusion criteria were advanced (FIGO Stage III & IV) high‐grade non‐mucinous ovarian cancer, diagnosed between 1 July 2023 and 30 June 2024. Patients with high‐grade mucinous, low‐grade epithelial, and non‐epithelial histotypes were excluded. The study setting was the sole public provider of gynecologic cancer services in Western Australia offering centralized care to a population of more than 1.4 million women. Western Australia is the largest state in Australia and covers an area of more than 2.6 million km^2^ [13]. The study endpoints were: completion and outcomes of HRD and germline genetic tests, and time taken to receive results.

Data were abstracted from electronic patient medical records, radiology reports, and molecular oncology laboratory databases at the Peter MacCallum Cancer Centre (Melbourne, Victoria) and PathWest (Nedlands, Western Australia), de‐identified, and entered in an Excel spreadsheet by the first author. The following variables were recorded: primary tumor site, histotype, stage, germline testing, tumor HRD testing and HRD result, date of HRD test request, date results reported, primary surgery or neoadjuvant chemotherapy, number of chemotherapy cycles prior to HRD testing, residual disease at surgery, chemotherapy response score (CRS), method of obtaining tumor tissue for HRD testing, type of tissue tested, estimated tumor purity, and patient consent for HRD testing.

The WAGCS is the sole public provider of gynecological cancer services in Western Australia, offering centralized care to a population of 1.435 million women. In the initial phases of implementing HRD testing in Western Australia, formalin‐fixed paraffin‐embedded tissue samples were sent to the molecular oncology laboratory at the Peter MacCallum Cancer Centre in Melbourne, Victoria, which used the SOPHiA DDM HRD solution panel (SOPHiA Genetics). In this assay, HRD status is represented by the genomic instability index (GII). A GII ≤ 0 was deemed HRD negative. By the third quarter of 2023, HRD testing was performed at the PathWest molecular oncology laboratory in Nedlands, Western Australia, using the Illumina TruSight Oncology 500 (TSO 500) targeted hybrid‐capture based next‐generation sequencing (NGS) panel, which is a comprehensive targeted NGS panel that determines the somatic mutation profile for 523 genes for single nucleotide variants, indels, and copy number abnormalities and 55 genes for fusion and splice variant detection. The HRD status is represented as a genomic instability score (GIS) calculated using the sum of three values—Loss of heterozygosity (LOH), Telomeric allelic imbalance (TAI) and Large‐Scale State transitions (LST's). A score of greater than or equal to 42 is considered HRD positive. Full details of the TSO 500 HRD workflow assay are available at [14].

Genomic DNA was extracted from formalin‐fixed paraffin‐embedded tissue obtained from image‐guided core biopsy or surgery. Ascitic fluid was first centrifuged to concentrate malignant cells and then fixed for paraffin embedding and sectioning. Tumor content was evaluated using haematoxylin and eosin (H&E) staining and containing ≥ 30% tumor cells.

**FIGURE 1:**
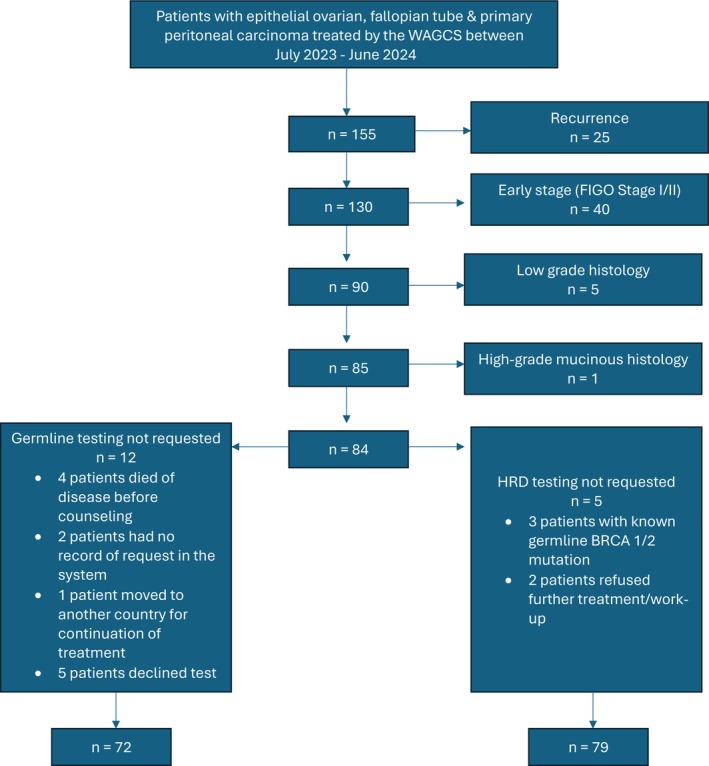
Flow chart of patients with the indication for HRD and germline testing and results. HRD = homologous recombination deficiency.

Germline testing was performed on blood samples for massively parallel sequencing on an Illumina NextSeq550 with Mid Output Kit v2.5 using an ovarian cancer panel that included *BRCA1, BRCA2, BRIP1, MLH1, MSH2, MSH6, PMS2, RAD51C, RAD51D* and truncating variants in *PALB2*. Parallel tumor and germline testing for patients with advanced high grade non‐mucinous epithelial ovarian cancer is standard of care in Western Australia (Figure 1) as per the British Gynaecological Cancer Society Consensus Statement [11].

### 2.1 Statistical Analysis

Data were analyzed using IBM SPSS Statistics for Windows, version 29 (IBM Corp., Armonk, N.Y., USA), with an alpha of 0.05 considered statistically significant. Categorical variables were described using frequency and percent. Continuous scale variables were described using mean and standard deviation and were assessed for normality by the Shapiro‐Wilk test. Nonparametric variables were described using median and interquartile range (IQR).

## 3 Results

**FIGURE 2:**
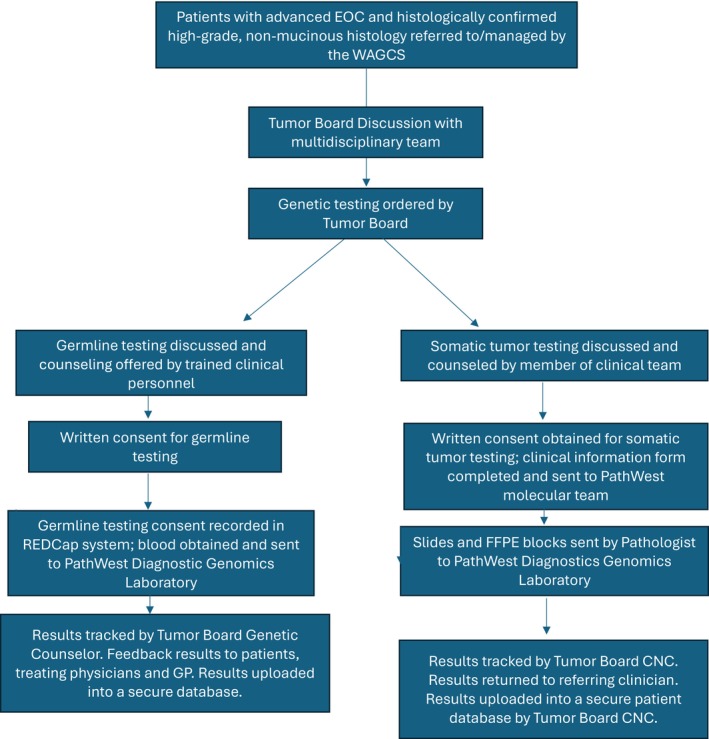
The process for parallel germline genetic and tumor testing for patients with advanced EOC managed by the WAGCS. EOC = epithelial ovarian cancer; FFPE = formalin‐foxed paraffin‐embedded; CNC, clinical nurse consultant. Counseling prior to germline testing was performed by trained members of the oncology team including gynecologic and medical oncologists, nursing staff and genetic counselors.

Between July 2023 and June 2024, the WAGCS had managed 155 patients with epithelial ovarian carcinoma. Within this group of patients, 25 had recurrent disease, 40 were newly diagnosed with Stage I and II disease, 5 had low‐grade histology, and one had high‐grade mucinous histology. The HRD test was indicated for the remaining 84 patients. Tumors from 5 patients were not tested for HRD (Figure 2), resulting in 79 patients undergoing HRD tumor testing (Figure 2).

A total of 50 of the 84 patients had FIGO stage IIIC, with less than half having primary debulking surgery. High‐grade serous carcinoma was the most common histotype (Table 1). The median duration between requesting HRD testing and receipt of results was 57 days (IQR 43 days) (Table 2). Out of the 84 patients eligible for the test, 5 of them did not have the test ordered (6%). Reasons for not testing included: three patients were known to carry BRCA1/2 germline mutations, and two patients declined testing.

**TABLE 1:** Patients' characteristics.

Characteristic	*n* = 84 (%)
Age at diagnosis, mean	69 (SD 11.24)
Stage of disease (FIGO 2014)	
3A2	3 (3.6)
3B	6 (7.1)
3C	50 (59.5)
4A	5 (6)
4B	20 (23.8)
Histopathology subtypes	
High grade serous carcinoma	75 (89.3)
Carcinosarcoma	5 (6)
High grade clear cell carcinoma	2 (2.4)
High grade seromucinous carcinoma	1 (1.2)
High grade hepatoid adenocarcinoma	1 (1.2)
Type of debulking surgery received	
Primary debulking surgery	39 (46.4)
Interval debulking surgery	19 (22.6)
Not debulked	23 (27.4)
Operated outside WAGCS	3 (3.6)

*Note:* FIGO 2014 – International Federation of Gynecology and Obstetrics 2014 staging system.

**TABLE 2:** Method of sample retrieval for HRD testing and results.

	*n* = 79 (%)
Method of obtaining sample for HRD testing	
Diagnostic laparoscopy	18 (22.8)
Image‐guided core biopsy	16 (20.3)
Image‐guided fine needle aspiration cytology (FNAC)	1 (1.3)
Aspiration of ascitic fluid/pleural fluid	11 (13.9)
Primary debulking surgery	14 (17.7)
Interval debulking surgery	11 (13.9)
Laparotomy & biopsy	7 (8.9)
Excision biopsy of lymph node	1 (1.3)
Time from HRD test to finalized report (median)	57 days
HRR test results	
HRD positive with sBRCAm	6 (7.6)
HRD positive without sBRCAm	23 (29.1)
HRD negative without sBRCAm	44 (55.7)
HRD negative with sBRCAm	3 (3.8)
Non‐diagnostic/inconclusive	3 (3.8)

Abbreviations: HRD, homologous recombination deficiency; sBRCAm, somatic BRCA mutation.

Of the 79 patients that had HRD testing ordered, three had non‐diagnostic/inconclusive results, all due to insufficient tumor quantity, with less than 30% of viable tumor tissue within these samples (Tables 2 and 3). One of these patients had the sample collected using a 20‐gauge core biopsy needle under image guidance, one patient following interval debulking surgery with a chemotherapy response score (CRS) of 2, and one following primary debulking surgery.

**TABLE 3:** Method of obtaining sample for HRD testing and outcomes.

Method of obtaining sample for HRD testing	HRD negative	HRD positive	Non‐diagnostic result
	*n* = 47	*n* = 29	*n* = 3
Diagnostic laparoscopy	11	7	0
Aspiration of ascitic fluid/pleural fluid	9	2	0
Image guided core biopsy			
Needle size			
16G	1	1	0
18G	7	3	0
20G	1	0	1
Not known	1	1	0
Image guided FNAC	1	0	0
Primary debulking surgery	7	6	1
Interval debulking surgery			
CRS 1	3	2	0
CRS 2	0	0	1
CRS 3	4	1	0
Laparotomy & biopsy/Excision biopsy	2	6	0

Abbreviations: CRS, chemotherapy response score; FNAC, fine needle aspiration cytology; HRD, homologous recombination deficiency.

Seventy‐six patients had an available GII/GIS and HRD was positive in 29 (38.2%) of them. A somatic *BRCA* mutation was detected in six of these 29 patients (20.7%) and HRD positive, BRCAwt was detected in 23 of 29 patients (79.3%). Of the six patients with HRD positive and tumor BRCA mutation, two had a concurrent germline BRCA mutation.

Tumor tissue from 7 patients was tested using the SOPHiA DDM HRD solution panel (SOPHiA Genetics) and the Illumina TruSight Oncology 500 (TSO 500) targeted hybrid‐capture based next generation sequencing (NGS) panel was used in 72 patients. HRD results were obtained for all 7 patients whose tumors were tested with the SOPHiA DDM HRD solution panel (SOPHiA Genetics).

Of the 47 patients with HRD negative results, three had a tumor *BRCA* mutation (GIS of 9, 28 and 31 respectively). These three patients had a co‐existing germline mutation as well. Eight patients with negative HRD results did not have germline testing done. The remaining 36 out of these 47 patients all had normal germline test results.

Out of the three patients with non‐diagnostic HRD test results, two had normal germline test results, and one patient died prior to testing.

Modes of tumor biopsy are shown in Table 2. There were 16 patients that had image‐guided core biopsy to obtain samples for histopathology and HRD testing. Core biopsy needle sizes 16G and 18G both produced 100% adequate samples for analysis. Whereas one of two patients that had a biopsy taken with a 20G needle had a non‐diagnostic HRD result (Table 3). Ten of 11 patients who were treated by neoadjuvant chemotherapy and whose biopsies were obtained at interval cytoreductive surgery had sufficient tumor tissue for testing (Table 3). All ascitic/pleural fluid samples sent for HRD testing yielded diagnostic results.

Nineteen patients received neoadjuvant chemotherapy and CRS was reported for 13 of these patients. Six patients' tumors had a CRS of 1 and two of these were HRD positive. Six patients' tumors had a CRS of 3, and one was HRD positive. One patient's tumor had a CRS of 2, and the tumor purity was insufficient for HRD testing.

Twenty‐three of 84 (27.4%) patients did not undergo debulking surgery. Reasons for this included disease progression during neoadjuvant chemotherapy (*n* = 9), unresectable disease (*n* = 2), deceased prior to planned treatment (*n* = 3), had treatment overseas (*n* = 1) and declined treatment (*n* = 8).

Of 84 eligible patients, 72 (85.7%) had germline testing. Of the 12 that were not tested, four patients had died before genetic counseling could be offered, two patients had no record of a germline testing request, one patient moved to another country for continuation of treatment, while the remaining five patients declined germline testing (Table 4). Sixty‐one of the 721 patients did not have any germline mutations. Five patients were noted to have germline *BRCA1* mutations, and three patients had *BRCA2* mutations. Two patients had a *BRIP1* mutation, while one patient was found to have a *RAD51D* mutation (Table 4). Eight of these 11 patients with germline mutations were found to be either HRD positive or had a tumor *BRCA1/2* mutation and were HRD negative. The remaining three of the 11 patients did not have tumor testing as the knowledge of germline mutation preceded their diagnosis of advanced ovarian malignancy. Twenty‐three of the 29 patients (79.3%) that were HRD positive did not have a germline mutation.

**TABLE 4:** Results of germline genetic testing.

Total number of patients eligible for germline testing	*N* = 84
Not performed	12
Died	4
No record of referral/request	2
Moved overseas	1
Declined	5
No germline mutation identified	61
Germline pathogenic mutation identified	11
*BRCA 1*	5
*BRCA 2*	3
*BRIP1*	2
*RAD 51D*	1

## 4 Discussion

This is a population‐based study that reports the real‐world experience of the first year of implementation of HRD testing at the statewide gynecologic cancer service in Western Australia. The uptake of tumor testing was 94% and uptake of germline testing was 85.7%, which are below the 95% recommended in international guidelines [11]. However, reasons for not testing included rapid disease progression and death before testing could be performed, and a small proportion of patients declining testing, which is consistent with previous studies [15, 16].

The median time from request of HRD testing to results was 57 days (range 15–122 days) which is an acceptable timeframe for patients undergoing primary debulking surgery as it would take an average of 126 days, assuming patients receive six three‐weekly cycles of chemotherapy, which would allow sufficient time for tumor testing results to guide decisions regarding maintenance PARPi. However, if the sample was sent following interval cytoreductive surgery, this timeframe might be suboptimal. It is notable that the range of time taken to receive HRD test results was large (15–122 days). Factors such as sample type (ascitic fluid vs. core biopsy vs. surgical specimens), timing of sample collection (pre‐treatment or following neoadjuvant chemotherapy), and gauge of core biopsy needle can affect the HRD test turnaround time.

Notably, tumor biopsies taken at diagnostic laparoscopy, laparotomy, and ascitic/pleural fluid resulted in the highest success rate in providing a diagnostic tumor HRD test result. All ascitic/pleural fluid samples sent for HRD testing yielded diagnostic results which are consistent with published data that showed a comparable performance between cell‐free tumor DNA (cftDNA) in ascitic fluid and tumor tissue‐based testing [17]. Somatic or germline *BRCA* gene mutations are often determinants of the HRD profile. The HRD status may also depend on mutations or promoter hypermethylation in other genes involved in the HR pathway. However, mutational status is dynamic and can change over time, restoring HR status in tumor cells. Exposure to platinum‐based chemotherapy may result in loss of hypermethylation, especially when the modifications are epigenetic [18, 19] and secondary reversion mutations that restore *BRCA1* or *BRCA2* function [20]. Although this group of patients is known to develop resistance to PARPi, sampling the tumor following exposure to chemotherapy may not truly reflect the patient's HR status and hence sampling should ideally be performed prior to systemic treatment.

Our findings are consistent with existing literature which has shown that biopsies obtained for tumor testing following exposure to chemotherapy are known to have a lower percentage of viable tumor tissue and provide a lower DNA yield compared to chemotherapy‐naïve tumor tissue, especially in samples with CRS 2–3 [11, 12, 21, 22, 23, 24].

In the current study, 100% of tumor samples obtained via 16G and 18G needles yielded diagnostic results, whereas one of two tumor samples obtained with a 20G needle was non‐diagnostic. Consistent with this, a recently published ISUOG/ESGO consensus statement recommends at least two 18G needles or wider should be utilized to attain sufficient material for somatic tumor testing [25, 26, 27]. Of note, the three patients in the study that had non‐diagnostic tumor test results had a tumor purity of < 30%. Each of these three patients had different methods of tumor biopsy for testing, including primary surgery, interval cytoreductive surgery following neoadjuvant chemotherapy with a CRS 2, and an image‐guided core biopsy with a 20G needle.

Interestingly, three patients were HRD negative based on their GIS, but were found to have a tumor *BRCA* mutation. It is estimated that up to 10% of patients with high‐grade epithelial ovarian cancers have a pathogenic *BRCA1/2* tumor mutation, yet their genomic instability scores are consistent with an HRD‐negative tumor [11, 28]. The biological mechanisms underlying this are unknown but may include promoter methylation that silences the wild‐type *BRCA* allele, leading to a functional loss‐of‐function phenotype without a complete deletion of the allele that is associated with a loss of heterozygosity (LOH) [29], intra‐tumoral heterozygous somatic *BRCA* mutations arising that could be a consequence rather than the cause of tumorigenesis [20, 30] monoallelic loss‐of‐function somatic *BRCA* variants that have a subtle haplo‐insufficient phenotype without severe HRD [31], *BRCA* reversion variants that are somatic mutations which restore the open reading frame of a germline mutant allele often by correcting frameshift or nonsense mutations [32, 33, 34] or patients with mosaic germline *BRCA* variants [35]. Such patients are thought to respond poorly to PARPi and require close surveillance during PARPi treatment [36, 37].

Clinical implementation of HRD testing is significantly impacted by crucial limitations, including differences in assay methodologies, their processing requirements and delivery logistics, inconsistent definitions of HRD status, and challenges in interpretation of results. These factors may limit the optimal selection of patients for PARPi therapy [38]. In clinical trials, HRD is typically measured by centralized next‐generation sequencing (NGS) assays, requiring outsourced analysis that can significantly affect the success of molecular testing in clinical practice. Accuracy and reproducibility are further challenged by the growing variety of commercial NGS assays for HRD evaluation, along with the lack of standardized protocols and data interpretation. In‐house HRD testing could help bridge the gap between molecular analysis and routine diagnostics and has been shown to be feasible, but achieving standardized workflows and harmonized results remains difficult [39].

There are important limitations to our study that should be acknowledged, including its retrospective design that may have introduced bias and the relatively small number of patients. A strength of the study is that the WAGCS is a centralized statewide service which ensures accurate case ascertainment.

## 5 Conclusion

We report on the first year of tumor testing for HRD status in patients with advanced high‐grade non‐mucinous epithelial ovarian cancer in Western Australia in conjunction with germline genetic testing. Compliance with HRD testing was high, and only three of 79 (3.8%) patients had non‐diagnostic results. Tumor biopsies taken at diagnostic laparoscopy, laparotomy, and ascitic/pleural fluid had the highest likelihood of yielding a diagnostic tumor HRD test result. HRD testing should ideally be performed on tissue obtained prior to chemotherapy.

## Author Contributions

K.P. conceptualization, methodology, formal analysis, investigation, writing – original draft. E.C. investigation, methodology, writing – review and editing. B.A. methodology, investigation, writing – review and editing. E.R.A. conceptualization, methodology, writing – review and editing. C.A. conceptualization, methodology, writing – review and editing. M.B. conceptualization, methodology, writing – review and editing. G.H. methodology, investigation, writing – review and editing. Y.L. conceptualization, methodology, writing – review and editing. T.M. conceptualization, methodology, writing – review and editing. G.R.K.A.M. conceptualization, methodology, writing – review and editing. C.B.N. methodology, investigation, writing – review and editing. N.D.P. conceptualization, methodology, writing – review and editing. P.A.C. conceptualization, methodology, formal analysis, investigation, writing – original draft, review and editing.

## Conflicts of Interest

P.A.C. declares speaker's honoraria from AstraZeneca, Merck Sharpe & Dohme, and GlaxoSmithKline; advisory board—AstraZeneca; and stock—Reliis Ltd. All other authors declare no conflicts of interest.

## Data Availability

The data that support the findings of this study are available from the corresponding author upon reasonable request and subject to relevant ethics committee approval.
